# Supplementary material for: Association of Phytophthora with Declining Vegetation in an Urban Forest Environment
Source: Microorganisms. 2020 Jun 29;8(7):973. doi: 10.3390/microorganisms8070973 (PMC7409110; doi:10.3390/microorganisms8070973)
Supplement: Supplementary file 1 [file microorganisms-08-00973-s001.pdf]

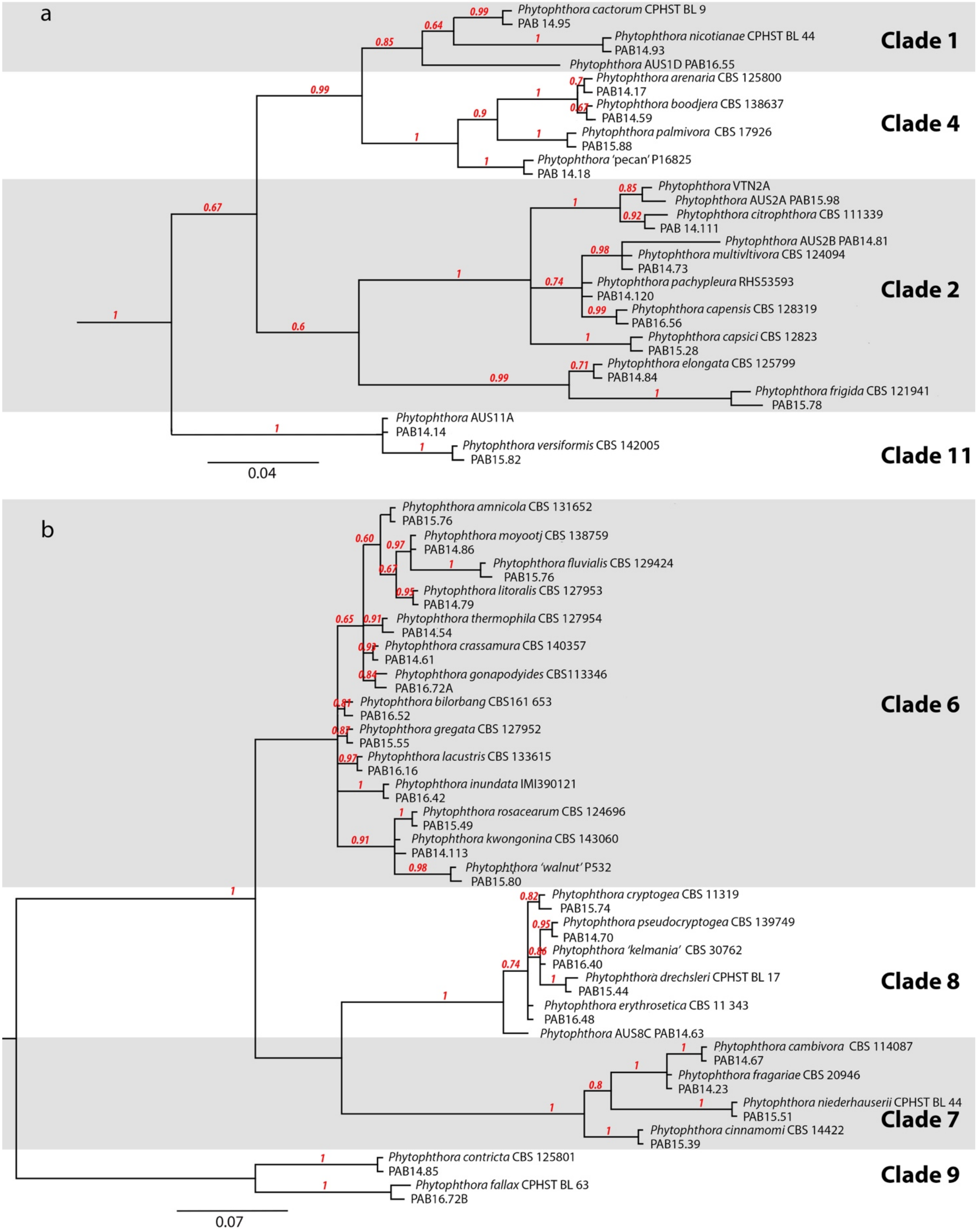

Figure S1. Bayesian tree based on ITS1 gene region containing representative sequences of all phylotypes found in the current study. For visualisation purposes and for the identification of MOTUs the phylogeny was divided into two separate analyses (a) Clades 1-4, and 11 (b) Clades 6-9. MOTUs were assigned to new phylotypes if they did not match any known Phytophthora species. Bayesian posterior probabilities are given above the branch.

Table S1. Variables measured for each of the sites sampled

SAMPLE	LOCATION	PARK CLASS	SOIL FAMILY	PARK AREA	CANOPY HEALTH	CANOPY COVER	PHYT_PA	NUMBER-PHYT
PAB 14-18	Aberdare Park	Open Space	LS1	11519.46	4.08	2162.25	1	5
PAB 14-87	Adelaide Park	Conservation Area	Karrakatta	2232.59	-6.42	520.50	1	2
PAB 14-86	Adelaide Park	Open Space	Karrakatta	20639.75	1.66	3921.75	1	5
PAB 16-12	Alfreton Reserve	Conservation Area	Karrakatta	18996.91	-8.00	5399.50	0	0
PAB 16-13	Alfreton Reserve	Open Space	Karrakatta	10691.64	-10.53	2244.50	0	0
PAB 15-105	Baltusrol Park	Open Space	Quindalup	19557.75	2.63	7939.25	1	6
PAB 15-103	Barridale Park	Park	Karrakatta	42039.84	10.77	9411.00	1	7
PAB 15-104	Barridale Park	Park	Karrakatta	42039.84	10.77	9411.00	1	3
PAB 14-90	Beaumaris Park	Conservation Area	Karrakatta	52769.25	19.51	6837.50	1	1
PAB 14-93	Beaumaris Park	Conservation Area	Karrakatta	52769.25	19.51	6837.50	1	7
PAB 16-57	Bethany Park	Conservation Area	Karrakatta	7066.89	4.09	1494.00	0	0
PAB 16-58	Bethany Park	Conservation Area	Karrakatta	7067.89	4.09	1494.00	0	0
PAB 16-59	Bethany Park	Conservation Area	Karrakatta	7067.89	4.09	1494.00	0	0
PAB 14-73	Blackboy Park	Open Space	Quindalup	20750.11	9.65	5612.25	1	1
PAB 14-74	Blackboy Park	Open Space	Quindalup	20750.11	9.65	5612.25	0	0
PAB 14-75	Blackboy Park	Open Space	Quindalup	20750.11	9.65	5612.25	1	1
PAB 15-96	Blue Lake Park	Conservation Area	Karrakatta	38697.31	2.65	10953.75	1	4
PAB 15-97	Blue Lake Park	Conservation Area	Karrakatta	38697.31	2.65	10953.75	1	6
PAB 15-98	Blue Lake Park	Conservation Area	Karrakatta	38697.31	2.65	10953.75	1	5
PAB 15-99	Blue Lake Park	Conservation Area	Karrakatta	38697.31	2.65	10953.75	1	9
PAB 15-33	Brazier Park	Open Space	Karrakatta	6719.00	8.07	1400.50	1	3
PAB 14-103	Burns Beach Park	Open Space	Karrakatta	393979.79	-1.73	13756.25	0	0
PAB 16-79	Burns Beach Park	Open Space	Karrakatta	393978.79	-1.73	13756.25	0	0
PAB 16-80	Burns Beach Park	Open Space	Quindalup	393979.79	-1.73	13756.25	0	0
PAB 14-45	Cadogen Reserve	Conservation Area	S7	622478.35	-2.14	165379.75	0	0
PAB 16-69	Caledonia Park	Park	Karrakatta	900.27	5.30	409.50	0	0
PAB 16-70	Caledonia Park	Park	Karrakatta	900.27	5.30	409.50	0	0
PAB 16-71	Caledonia Park	Park	Karrakatta	900.27	5.30	409.50	0	0
PAB 16-76	Callender Park	Open Space	Karrakatta	10483.24	9.29	581.50	0	0
PAB 16-77	Callender Park	Open Space	Karrakatta	10483.24	9.29	581.50	0	0
PAB 16-33	Camberwarra Park	Open Space	Karrakatta	23056.61	12.89	6272.50	0	0
PAB 16-64	Carnaby Reserve	Conservation Area	Karrakatta	21733.29	3.17	2169.75	0	0
PAB 16-65	Carnaby Reserve	Conservation Area	Karrakatta	21733.29	3.17	2169.75	0	0
PAB 15-49	Cawarra Park	Conservation Area	Quindalup	35201.49	2.27	4148.50	1	5
PAB 15-50	Cawarra Park	Conservation Area	Quindalup	35201.49	2.27	4148.50	1	5
PAB 14-114	Central Park	Conservation Area	Karrakatta	30012.47	2.91	1266.25	0	0
PAB 14-115	Central Park	Open Space	Karrakatta	41644.07	5.91	11249.00	0	0
PAB 16-24	Chadlington Reserve	Conservation Area	Karrakatta	8778.61	4.49	1525.75	1	4
PAB 16-24	Chadlington Reserve	Conservation Area	Karrakatta	8778.61	4.49	1525.75	1	6
PAB 16-25	Chadlington Reserve	Conservation Area	Karrakatta	8778.61	4.49	1525.75	0	0
PAB 16-26	Chadlington Reserve	Conservation Area	Karrakatta	8778.61	4.49	1525.75	0	0
PAB 15-44	Chichester Park	Open Space	Spearwood	28423.99	-2.44	14237.25	1	7
PAB 15-45	Chichester Park	Open Space	Spearwood	28423.99	-2.44	14237.25	1	6
PAB 15-46	Chichester Park	Park	Spearwood	63127.49	-2.54	6810.00	1	6
PAB 14-101	Clermont Park	Conservation Area	Karrakatta	15855.41	4.84	400.50	1	1
PAB 14-102	Clermont Park	Conservation Area	Karrakatta	15855.41	4.84	1044.25	1	1

SAMPLE	LOCATION	PARK CLASS	SOIL FAMILY	PARK AREA	CANOPY HEALTH	CANOPY COVER	PHYT_PA	NUMBER-PHYT
PAB 14-100	Clermont Park	Open Space	Karrakatta	3761.33	6.74	1044.25	0	0
PAB 16-14	Conidae Park	Open Space	Karrakatta	15270.92	17.05	3228.00	0	0
PAB 16-16	Conidae Park	Open Space	Karrakatta	15270.92	17.05	3228.00	1	6
PAB 14-78	Craigie Open Space	Conservation Area	Karrakatta	559906.19	-6.09	170592.25	1	9
PAB 14-79	Craigie Open Space	Conservation Area	Karrakatta	559906.19	-6.09	170592.25	1	8
PAB 14-80	Craigie Open Space	Conservation Area	Karrakatta	559906.19	-6.09	170592.25	1	10
PAB 14-81	Craigie Open Space	Conservation Area	Karrakatta	559906.19	-6.09	170592.25	1	2
PAB 14-82	Craigie Open Space	Conservation Area	Karrakatta	559906.19	-6.09	170592.25	1	2
PAB 14-83	Craigie Open Space	Conservation Area	Karrakatta	559906.19	-6.09	170592.25	1	6
PAB 14-84	Craigie Open Space	Conservation Area	Karrakatta	559906.19	-6.09	170592.25	1	14
PAB 14-85	Craigie Open Space	Conservation Area	Karrakatta	559906.19	-6.09	170592.25	1	19
PAB 15-100	Cranston Park	Conservation Area	Karrakatta	28054.33	-17.42	386.75	1	6
PAB 15-101	Cranston Park	Conservation Area	Karrakatta	28054.33	-17.42	386.75	1	2
PAB 16-73	Earlsferry Park	Open Space	Karrakatta	7246.56	1.54	766.25	0	0
PAB 15-81	Emerald Park	Park	Karrakatta	63682.48	11.39	11800.75	1	6
PAB 15-82	Emerald Park	Park	Karrakatta	63682.48	11.39	11800.75	1	7
PAB 15-83	Emerald Park	Park	Karrakatta	63682.48	11.39	11800.75	1	9
PAB 15-84	Emerald Park	Park	Karrakatta	63682.48	11.39	11800.75	1	9
PAB 14-111	Fairway Park	Conservation Area	Karrakatta	6962.14	6.01	264.75	1	8
PAB 14-112	Fairway Park	Conservation Area	Karrakatta	6962.14	6.01	264.75	0	0
PAB 15-34	Fernwood Park	Open Space	Karrakatta	3224.84	-4.82	837.50	1	3
PAB 15-79	Garrong Park	Conservation Area	Spearwood	4510.52	-2.00	2449.50	1	7
PAB 15-80	Garrong Park	Conservation Area	Spearwood	4510.52	-2.00	2449.50	1	10
PAB 15-35	Gibson Park	Open Space	Quindalup	37050.18	8.21	9535.75	1	4
PAB 15-36	Gibson Park	Open Space	Quindalup	37050.18	8.21	9535.75	1	6
PAB 16-07	Glenbar Park	Conservation Area	S7	53289.54	8.31	6251.50	0	0
PAB 16-08	Glenbar Park	Conservation Area	LS1	53289.54	8.31	6251.50	0	0
PAB 16-09	Glenbar Park	Conservation Area	LS1	53289.54	8.31	6251.50	0	0
PAB 14-12	Granadilla Park	Open Space	LS1	37863.38	11.00	8067.75	1	4
PAB 14-14	Granadilla Park	Open Space	LS1	37863.38	11.00	8067.75	1	2
PAB 14-15	Granadilla Park	Open Space	LS1	37863.38	11.00	8067.75	1	1
PAB 15-89	Greenshank Park	Open Space	Karrakatta	4177.94	8.31	49.25	1	6
PAB 15-90	Greenshank Park	Open Space	Karrakatta	4177.94	8.31	1109.75	1	5
PAB 15-88	Greenshank Park	Thoroughfare	Karrakatta	486.88	13.08	1109.75	1	8
PAB 14-16	Hawker Park	Conservation Area	S7	12070.96	-0.96	4257.75	0	0
PAB 14-17	Hawker Park	Conservation Area	LS1	12070.96	-0.96	4257.75	1	1
PAB 14-21	Hawker Park	Park	S7	34634.67	10.77	3950.25	0	0
PAB 14-54	Hepburn Heights	Conservation Area	Karrakatta	221807.93	0.93	20792.00	1	2
PAB 14-55	Hepburn Heights	Conservation Area	Karrakatta	221807.93	0.93	20792.00	1	3
PAB 14-56	Hepburn Heights	Conservation Area	Karrakatta	221807.93	0.93	20792.00	1	6
PAB 14-57	Hepburn Heights	Conservation Area	Karrakatta	221807.93	0.93	20792.00	0	0
PAB 14-58	Hepburn Heights	Conservation Area	Karrakatta	221807.93	0.93	20792.00	1	5
PAB 14-59	Hepburn Heights	Conservation Area	Karrakatta	221807.93	0.93	20792.00	1	2
PAB 14-60	Hepburn Heights	Conservation Area	Karrakatta	221807.93	0.93	20792.00	1	21
PAB 14-66	Hillarys Beach Park	Open Space	Quindalup	60156.77	4.64	6470.00	0	0
PAB 14-67	Hillarys Beach Park	Open Space	Quindalup	60156.77	4.64	6470.00	1	8
PAB 14-63	Hillarys Foreshore	Conservation Area	Quindalup	618427.13	0.52	92001.00	1	15
PAB 14-64	Hillarys Foreshore	Conservation Area	Quindalup	618427.13	0.52	92001.00	1	1

SAMPLE	LOCATION	PARK CLASS	SOIL FAMILY	PARK AREA	CANOPY HEALTH	CANOPY COVER	PHYT_PA	NUMBER-PHYT
PAB 14-65	Hillarys Foreshore	Conservation Area	Quindalup	618427.13	0.52	92001.00	1	5
PAB 14-68	Hillarys Foreshore	Conservation Area	Quindalup	618427.13	0.52	92001.00	1	2
PAB 14-69	Hillarys Foreshore	Conservation Area	Quindalup	618427.13	0.52	92001.00	1	1
PAB 14-70	Hillarys Foreshore	Conservation Area	Quindalup	618427.13	0.52	92001.00	1	5
PAB 14-71	Hillarys Foreshore	Conservation Area	Quindalup	618427.13	0.52	92001.00	1	2
PAB 14-72	Hillarys Foreshore	Conservation Area	Quindalup	618427.13	0.52	92001.00	1	12
PAB 16-35	Hillarys Foreshore	Conservation Area	Quindalup	618427.13	0.52	92001.00	1	7
PAB 16-36	Hillarys Foreshore	Conservation Area	Quindalup	618427.13	0.52	92001.00	0	0
PAB 16-37	Hillarys Foreshore	Conservation Area	Quindalup	618427.13	0.52	92001.00	0	0
PAB 15-30	Hillarys Park	Park	Quindalup	70649.30	0.09	7843.50	1	4
PAB 15-31	Hillarys Park	Park	Quindalup	70649.30	0.09	7843.50	0	0
PAB 15-32	Hillarys Park	Park	Quindalup	70649.30	0.09	7843.50	1	5
PAB 16-61	Huntingdale Park	Open Space	Karrakatta	9101.86	-5.87	1592.25	0	0
PAB 16-62	Huntingdale Park	Open Space	Karrakatta	9101.86	-5.87	1592.25	0	0
PAB 16-63	Huntingdale Park	Open Space	Karrakatta	9101.86	-5.87	1592.25	0	0
PAB 16-53	Iluka Reserve	Conservation Area	Karrakatta	362177.28	3.04	4604.00	1	4
PAB 16-54	Iluka Reserve	Conservation Area	Karrakatta	362177.28	3.04	4604.00	1	4
PAB 16-55	Iluka Reserve	Conservation Area	Quindalup	362178.28	3.04	4604.00	1	16
PAB 16-56	Iluka Reserve	Conservation Area	Quindalup	362177.28	3.04	4604.00	1	15
PAB 15-20	Juniper Park	Park	S7	47055.09	10.69	24345.50	1	6
PAB 15-21	Juniper Park	Park	S7	47055.09	10.69	24345.50	1	7
PAB 15-22	Juniper Park	Park	S7	47055.09	10.69	24345.50	0	0
PAB 16-38	Kallaroo Foreshore	Conservation Area	Quindalup	245679.59	0.30	6780.50	1	6
PAB 16-39	Kallaroo Foreshore	Conservation Area	Quindalup	245679.59	0.30	6780.50	0	0
PAB 16-40	Kallaroo Foreshore	Conservation Area	Quindalup	245679.59	0.30	6780.50	1	6
PAB 16-41	Kallaroo Foreshore	Conservation Area	Quindalup	245679.59	0.30	6780.50	1	12
PAB 16-42	Kallaroo Foreshore	Conservation Area	Quindalup	245679.59	0.30	6780.50	1	7
PAB 16-43	Kallaroo Park	Conservation Area	Karrakatta	22839.23	0.10	1001.50	1	1
PAB 16-44	Kallaroo Park	Conservation Area	Karrakatta	22839.23	0.10	1001.50	1	5
PAB 16-45	Kallaroo Park	Conservation Area	Karrakatta	22839.23	0.10	1001.50	1	6
PAB 16-46	Kallaroo Park	Conservation Area	Karrakatta	22839.23	0.10	1001.50	0	0
PAB 16-47	Kallaroo Park	Conservation Area	Karrakatta	22839.23	0.10	1001.50	1	4
PAB 16-27	Kingsley Access Path	Thoroughfare	Karrakatta	44781.51	10.04	14286.00	1	7
PAB 16-20	Korella Park	Conservation Area	Quindalup	30785.72	4.78	12629.75	0	0
PAB 16-21	Korella Park	Park	Quindalup	15517.47	12.59	1650.00	0	0
PAB 16-22	Korella Park	Conservation Area	Quindalup	30785.72	4.78	12629.75	0	0
PAB 16-23	Korella Park	Conservation Area	Quindalup	30786.72	4.78	12629.75	1	1
PAB 16-74	Lady Evelyn Park	Conservation Area	Karrakatta	7002.89	7.90	835.75	0	0
PAB 14-113	Lakeside Park	Conservation Area	Karrakatta	31343.63	0.35	3552.25	1	3
PAB 14-117	Lakevalley Park	Conservation Area	Quindalup	14504.65	-2.20	2772.00	0	0
PAB 14-118	Lakevalley Park	Conservation Area	Karrakatta	14504.65	-2.20	2772.00	1	1
PAB 15-37	Legana Park	Open Space	Spearwood	35380.23	7.58	15565.50	1	4
PAB 15-38	Legana Park	Open Space	Spearwood	35380.23	7.58	15565.50	1	4
PAB 14-120	Lexcen Park	Conservation Area	Quindalup	21798.97	13.97	8508.75	1	5
PAB 14-61	Lilburne Park	Conservation Area	Karrakatta	56793.33	2.75	13487.00	1	1
PAB 14-62	Lilburne Park	Conservation Area	Karrakatta	56793.33	2.75	13487.00	0	0
PAB 15-65	Littorina Park	Conservation Area	Quindalup	32327.81	17.33	1801.75	1	3
PAB 15-67	Littorina Park	Conservation Area	Quindalup	32327.81	17.33	4133.00	1	6

SAMPLE	LOCATION	PARK CLASS	SOIL FAMILY	PARK AREA	CANOPY HEALTH	CANOPY COVER	PHYT_PA	NUMBER-PHYT
PAB 15-68	Littorina Park	Conservation Area	Quindalup	32327.81	17.33	1801.75	1	9
PAB 15-69	Littorina Park	Conservation Area	Quindalup	32327.81	17.33	1801.75	1	7
PAB 15-66	Littorina Park	Park	Quindalup	28099.51	18.78	1801.75	1	6
PAB 16-60	Lysander Reserve	Open Space	Karrakatta	5865.57	2.88	721.00	0	0
PAB 16-75	Manapouri Park	Open Space	Karrakatta	4095.52	8.87	696.75	0	0
PAB 14-76	Maritana Park	Conservation Area	Quindalup	31099.89	0.72	1804.00	1	5
PAB 14-77	Maritana Park	Conservation Area	Quindalup	31099.89	0.72	1804.00	1	9
PAB 16-34	Maritana Park	Conservation Area	Quindalup	31099.89	0.72	1804.00	1	11
PAB 16-78	Menteith Park	Open Space	Karrakatta	10348.35	14.26	1692.25	0	0
PAB 15-91	Nanika Park	Conservation Area	Karrakatta	15254.13	7.34	1694.00	1	3
PAB 15-92	Nanika Park	Conservation Area	Karrakatta	15254.13	7.34	1694.00	1	7
PAB 15-93	Nanika Park	Conservation Area	Karrakatta	15254.13	7.34	1694.00	1	6
PAB 15-94	Nanika Park	Conservation Area	Karrakatta	15254.13	7.34	1694.00	1	4
PAB 15-95	Nanika Park	Conservation Area	Karrakatta	15254.13	7.34	1694.00	1	1
PAB 14-94	Naturaliste Park	Conservation Area	Karrakatta	33107.78	1.28	1850.75	0	0
PAB 14-95	Naturaliste Park	Conservation Area	Karrakatta	33107.78	1.28	1850.75	1	3
PAB 14-96	Naturaliste Park	Conservation Area	Karrakatta	33107.78	1.28	1850.75	1	2
PAB 14-104	Negresco Park	Open Space	Karrakatta	2700.11	-0.56	712.50	1	2
PAB 16-48	Ocean Reef Foreshore	Conservation Area	Quindalup	1079298.87	2.97	15676.00	1	4
PAB 16-49	Ocean Reef Foreshore	Conservation Area	Quindalup	1079298.87	2.97	15676.00	1	8
PAB 16-50	Ocean Reef Foreshore	Conservation Area	Quindalup	1079298.87	2.97	15676.00	1	3
PAB 16-51	Ocean Reef Foreshore	Conservation Area	Karrakatta	1079298.87	2.97	15676.00	1	1
PAB 16-52	Ocean Reef Foreshore	Conservation Area	Karrakatta	1079298.87	2.97	15676.00	1	6
PAB 15-59	Periwinkle Park	Conservation Area	Karrakatta	37308.50	3.15	8790.25	1	4
PAB 15-60	Periwinkle Park	Conservation Area	Karrakatta	37308.50	3.15	8790.25	1	3
PAB 15-61	Periwinkle Park	Conservation Area	Karrakatta	37308.50	3.15	8790.25	1	7
PAB 15-62	Periwinkle Park	Conservation Area	Karrakatta	37308.50	3.15	8790.25	1	6
PAB 15-63	Periwinkle Park	Conservation Area	Karrakatta	37308.50	3.15	8790.25	1	6
PAB 15-64	Periwinkle Park	Conservation Area	Karrakatta	37308.50	3.15	8790.25	1	5
PAB 16-72	Pine valley	Conservation Area	Karrakatta	21970.47	-0.40	3100.25	1	12
PAB 15-77	Plumdale Park	Open Space	Karrakatta	17229.56	-4.64	1826.25	1	10
PAB 15-78	Plumdale Park	Open Space	Karrakatta	17229.56	-4.64	1826.25	1	5
PAB 15-28	Porteous Park	Conservation Area	Karrakatta	16124.34	-6.22	493.00	1	8
PAB 15-29	Porteous Park	Conservation Area	Karrakatta	16124.34	-6.22	493.00	1	7
PAB 16-17	Quarry Ramble Park	Conservation Area	Karrakatta	18735.47	0.92	9462.50	0	0
PAB 16-19	Quarry Ramble Park	Conservation Area	Karrakatta	18735.47	0.92	2181.75	0	0
PAB 16-18	Quarry Ramble Park	Open Space	Spearwood	15040.54	13.08	9462.50	0	0
PAB 16-67	Riversdale Park	Open Space	Karrakatta	5428.98	-0.50	684.25	0	0
PAB 16-68	Riversdale Park	Open Space	Karrakatta	5428.98	-0.50	2474.75	0	0
PAB 16-66	Riversdale Park	Open Space	Karrakatta	3516.12	6.55	2474.75	0	0
PAB 16-28	Robertson Road	Thoroughfare	Karrakatta	44781.51	10.04	14286.00	0	0
PAB 16-29	Robertson Road	Thoroughfare	Karrakatta	44781.51	10.04	14286.00	0	0
PAB 16-32	Robertson Road	Thoroughfare	Karrakatta	44781.51	10.04	14286.00	0	11
PAB 15-74	Sandalford Park	Conservation Area	Karrakatta	7908.12	-4.34	7984.50	1	6
PAB 15-75	Sandalford Park	Conservation Area	Karrakatta	7908.12	-4.34	7984.50	1	4
PAB 15-76	Sandalford Park	Conservation Area	Karrakatta	7908.12	-4.34	7984.50	1	9
PAB 15-70	Sandalford Park	Open Space	Karrakatta	36312.28	9.79	7984.50	1	10
PAB 15-71	Sandalford Park	Open Space	Karrakatta	36312.28	9.79	2575.75	1	7

SAMPLE	LOCATION	PARK CLASS	SOIL FAMILY	PARK AREA	CANOPY HEALTH	CANOPY COVER	PHYT_PA	NUMBER-PHYT
PAB 15-72	Sandalford Park	Open Space	Karrakatta	36312.28	9.79	2575.75	1	4
PAB 15-73	Sandalford Park	Open Space	Karrakatta	36312.28	9.79	2575.75	1	6
PAB 14-47	Shepherds Bush	Conservation Area	Karrakatta	160651.58	2.70	30854.50	1	4
PAB 14-48	Shepherds Bush	Conservation Area	Karrakatta	160651.58	2.70	30854.50	0	0
PAB 14-51	Shepherds Bush	Conservation Area	Karrakatta	160651.58	2.70	30854.50	1	3
PAB 14-52	Shepherds Bush	Conservation Area	Karrakatta	160651.58	2.70	30854.50	1	1
PAB 16-30	Shephards Bush	Conservation Area	Karrakatta	160651.58	2.70	30854.50	0	0
PAB 16-31	Shephards Bush	Conservation Area	Karrakatta	160651.58	2.70	30854.50	1	8
PAB 14-98	James McCusker Park	Conservation Area	Karrakatta	34205.31	12.30	3818.75	1	1
PAB 14-99	James McCusker Park	Conservation Area	Karrakatta	34205.31	12.30	3818.75	1	8
PAB 15-85	St Clair Park	Conservation Area	Karrakatta	35692.63	3.90	4340.75	1	11
PAB 15-86	St Clair Park	Conservation Area	Karrakatta	35692.63	3.90	4340.75	1	7
PAB 15-87	St Clair Park	Conservation Area	Karrakatta	35692.63	3.90	4340.75	1	6
PAB 15-41	Timberlane Park	Conservation Area	Karrakatta	26232.90	0.63	7919.75	1	3
PAB 15-40	Timberlane Park	Park	Spearwood	53547.08	6.57	11644.00	1	8
PAB 15-42	Timberlane Park	Park	Spearwood	40378.16	6.57	7919.75	1	4
PAB 15-52	Tom Simpson Park	Conservation Area	Quindalup	40378.16	-8.05	1607.75	1	4
PAB 15-55	Tom Simpson Park	Conservation Area	Quindalup	40378.16	-8.05	1607.75	1	9
PAB 15-56	Tom Simpson Park	Conservation Area	Quindalup	40378.16	-8.05	1607.75	1	5
PAB 15-58	Tom Simpson Park	Conservation Area	Quindalup	40378.16	-8.05	1607.75	1	8
PAB 15-51	Tom Simpson Park	Open Space	Quindalup	40378.16	-8.05	1607.75	1	9
PAB 15-53	Tom Simpson Park	Open Space	Quindalup	40378.16	-8.05	1607.75	1	6
PAB 15-54	Tom Simpson Park	Open Space	Quindalup	40378.16	-8.05	1607.75	1	5
PAB 15-57	Tom Simpson Park	Open Space	Quindalup	40378.16	-8.05	1607.75	1	7
PAB 15-43	Trappers Park	Conservation Area	Karrakatta	20217.03	6.86	8242.00	1	5
PAB 15-23	Trigonometric Park	Conservation Area	LS1	26248.50	-2.43	2915.25	1	7
PAB 15-24	Trigonometric Park	Conservation Area	LS1	26248.50	-2.43	2915.25	1	7
PAB 15-25	Trigonometric Park	Conservation Area	LS1	26248.50	-2.43	2915.25	1	6
PAB 15-26	Trigonometric Park	Conservation Area	LS1	26248.50	-2.43	2915.25	1	3
PAB 15-27	Trigonometric Park	Conservation Area	LS1	26248.50	-2.43	2915.25	1	4
PAB 15-47	Warrandyte Park	Park	Quindalup	72200.40	4.21	3348.00	1	5
PAB 14-19	Warwick Open Space	Conservation Area	S7	622478.35	-2.14	165379.75	1	5
PAB 14-22	Warwick Open Space	Conservation Area	S7	622478.35	-2.14	165379.75	1	1
PAB 14-23	Warwick Open Space	Conservation Area	S7	622478.35	-2.14	165379.75	1	10
PAB 14-24	Warwick Open Space	Conservation Area	S7	622478.35	-2.14	165379.75	1	6
PAB 14-25	Warwick Open Space	Conservation Area	S7	622478.35	-2.14	165379.75	1	17
PAB 14-26	Warwick Open Space	Conservation Area	S7	622478.35	-2.14	165379.75	1	1
PAB 14-29	Warwick Open Space	Conservation Area	S7	622478.35	-2.14	165379.75	1	1
PAB 14-35	Warwick Open Space	Conservation Area	S7	622478.35	-2.14	165379.75	1	2
PAB 14-37	Warwick Open Space	Conservation Area	S7	622478.35	-2.14	165379.75	1	1
PAB 14-38	Warwick Open Space	Conservation Area	S7	622478.35	-2.14	165379.75	1	2
PAB 14-40	Warwick Open Space	Conservation Area	S7	622478.35	-2.14	165379.75	1	1
PAB 14-42	Warwick Open Space	Conservation Area	S7	622478.35	-2.14	165379.75	1	4
PAB 14-43	Warwick Open Space	Conservation Area	S7	622478.35	-2.14	165379.75	0	0
PAB 14-44	Warwick Open Space	Conservation Area	S7	622478.35	-2.14	165379.75	0	0
PAB 15-39	Waterview Park	Open Space	Spearwood	3748.19	4.23	1459.50	1	8

Table S2. Data matrix for all sampling sites from which *Phytophthora* was detected giving the number of reads for each of the 44 *Phytophthora* species.

Sample	CAC	NIC	ID	2A	2B	CAP	CAPS	CIT	ELO	FRI	MULT	PAC	ARE	BOO	PAL	PEC	AMN	BIL	CRA	FLU	GON	GRE	INU	KWO	LAC	LIT	MOY	ROS	WAL	THE	CAM	CIN	FRA	NIE	CRY	DRE	ERY	PSE	KEL	8C	CON	FAL	VER	11A
PAB 16-52			441								19							692												23									8					
PAB 16-53		2									3																			3									538					
PAB 16-54						15					159				2		36																					156						
PAB 16-35				4							76		11					13												26								62						
PAB 16-55		3	447	9		438					66		18				8	3				4	12					2		3	3	5	3					41						
PAB 16-56		37	28	53		55					295		80	70	8		2	10				43	40			2				26		2			3			245						
PAB 16-41		9		16							177		9	14	8		6	20				7	6							27								90						
PAB 16-49			188	4							52		3					14					5							6								32						
PAB 14-70					2						597		211											113														466						
PAB 14-63		49			2	125			4	3	374		7				157									64	40			3		86						64		135	22		7	
PAB 14-72		6			7	96					1006		24				48					2					10	9		9		26						92						
PAB 16-40		2									21				4								3															8	325					
PAB 14-69			1022																																									
PAB 16-42		2									94		20				6						20							23								40						
PAB 14-64		1034																																										
PAB 14-65											685								86				22															201		27				
PAB 14-68											863																											181						
PAB 14-71																			2989																			2						
PAB 16-38		899									106		8					25					17							12								53						
PAB 16-48											31																			21							11		525					
PAB 16-50													60																									8	420					
PAB 14-67		49				165					323		287		3		336														185							132						
PAB 14-25		42			2	70				2	754	18	36	26			83						20	2						63		63	29				8	85			4		28	
PAB 14-26					2						2017																																	
PAB 14-24					28									13																18		3												
PAB 14-17													1858																															
PAB 15-23		14									255		404				19											2				88			86									
PAB 15-24											62		695				92					2								105		120						104						
PAB 15-25											37		26		25		131															256						296						

Sample	CAC	NIC	ID	2A	2B	CAP	CAPS	CIT	ELO	FRI	MULT	PAC	ARE	BOO	PAL	PEC	AMN	BIL	CRA	FLU	GON	GRE	INU	KWO	LAC	LIT	MOY	ROS	WAL	THE	CAM	CIN	FRA	NIE	CRY	DRE	ERY	PSE	KEL	8C	CON	FAL	VER	11A
PAB 15-26											560											20								13														
PAB 15-27											197		43																			261						65					29	
PAB 14-19						809					511						237						85									194												
PAB 14-22											2280																																	
PAB 14-23		30				394					211		116				22						30							60		53	231					96						
PAB 14-29											2822																																	
PAB 14-35											2338																																	
PAB 14-37											2787																																	
PAB 14-38																												928		334								533						
PAB 14-40											1683																																	
PAB 14-42						543					486	30	2																									39						
PAB 15-98		153		403										357				44	156																									
PAB 16-24		22		4							194		6	7	12			41					69							12		16						187						
PAB 16-31				11							152			15	5			41												77		12						158						
PAB 14-85		62			2	27		60			849		114				138						25	55			30	111		52	7	145	9			6		28			44		20	
PAB 15-76		109			3			180			269		43				525							103						26		35												
PAB 15-28					129		92					92		105										18						82		93						16						
PAB 14-99		47			225			89					30				33													13		16						211						
PAB 14-111			129		696			167					18				314										91	54										141			22			
PAB 14-98					922																																							
PAB 14-81					4039																							2				1881												
PAB 14-54																														1461														
PAB 14-55						118					706			42			35															170												
PAB 15-29											360	17	177				36							8								72												
PAB 15-85		200						64		28	662				21		93							38			8			85		18						41						
PAB 15-86		73						57			625		87	29			230													35														
PAB 15-87		69				47		10			398		80				2															59												

Sample	CAC	NIC	ID	2A	2B	CAP	CAPS	CIT	ELO	FRI	MULT	PAC	ARE	BOO	PAL	PEC	AMN	BIL	CRA	FLU	GON	GRE	INU	KWO	LAC	LIT	MOY	ROS	WAL	THE	CAM	CIN	FRA	NIE	CRY	DRE	ERY	PSE	KEL	8C	CON	FAL	VER	11A
PAB 15-91		235											2																	281														
PAB 15-92											47		17	129			49										101			57								140						
PAB 15-93		150									731		15				44		125													78												
PAB 16-72	59	33									47		36					19			9	47	7	12										88				61				26		0
PAB 14-101																	599																											
PAB 14-102													645																															
PAB 14-113																								180																			2620	2
PAB 14-118													361																															
PAB 14-47						1735					265	154					2																											
PAB 14-51											390		125																									740						
PAB 14-52											830																																	
PAB 14-56											1016		8				71									49												168						
PAB 14-58											1122		13															13										7			26			
PAB 14-59											35			1371																														
PAB 14-60		28				162		3	6		736		32	19			97		5				15		6	34	8	11		66	26	189	2					125			10		29	
PAB 14-61																			1585																									
PAB 14-78						28		31			482		9													100	178	49					24					276						
PAB 14-79						691					203	2	180				163									205												19			176			
PAB 14-80						283		420			413		2				20									68	55					14						59			4			
PAB 14-82																																						1556						
PAB 14-83											868		11				142							130						277		243												
PAB 14-84		59				131			20		781		70				47							3		138	193	32		7		8						145			94		29	
PAB 14-87											483				261																													
PAB 14-90																																						599						
PAB 14-93		92						356			638		209				499									103	2																	
PAB 14-95	106																																					1702						
PAB 14-96		4																													1108													

Sample	CAC	NIC	ID	2A	2B	CAP	CAPS	CIT	ELO	FRI	MULT	PAC	ARE	BOO	PAL	PEC	AMN	BIL	CRA	FLU	GON	GRE	INU	KWO	LAC	LIT	MOY	ROS	WAL	THE	CAM	CIN	FRA	NIE	CRY	DRE	ERY	PSE	KEL	8C	CON	FAL	VER	11A
PAB 15-100		158									68		111				64															102						443						
PAB 15-101			3881								315																																	
PAB 15-41		2															110															259												
PAB 15-43											52		385				61															104						174						
PAB 15-59											927		208											85									55												
PAB 15-60											1243		59																				363												
PAB 15-61		215						60			348		220				115															98						138						
PAB 15-62											684		225				173							12						176		33												
PAB 15-63											579	165	15											31								130						33						
PAB 15-64											62		40				32															297						22						
PAB 15-74		61									317		25																			218			100			39						
PAB 15-75											639		108				148															155												
PAB 15-94		14									109						15																					11					214	
PAB 15-95		137									672																					282												
PAB 15-96		60									571		17				284			68																									
PAB 15-97		145				14					296		19																			59						71						
PAB 15-99		197									273	26	88		11		134															276						25					60	
PAB 16-43		889																																										
PAB 16-45		3									2						8					45																422						
PAB 16-47													260	465																								26	9					
PAB 16-34		4		5							191			8			7	13				16	5							24		20						119						
PAB 14-77		59			4						535	8	14	30			20													4								662						
PAB 15-69		180			4						698		105				340							2								156												
PAB 14-120					1435				2			1134					877																											
PAB 14-76						61					832		18				6										107											583						
PAB 15-49											151						217							23				115		16													
PAB 15-50		120									522		33				14																						7					

Sample	CAC	NIC	ID	2A	2B	CAP	CAPS	CIT	ELO	FRI	MULT	PAC	ARE	BOO	PAL	PEC	AMN	BIL	CRA	FLU	GON	GRE	INU	KWO	LAC	LIT	MOY	ROS	WAL	THE	CAM	CIN	FRA	NIE	CRY	DRE	ERY	PSE	KEL	8C	CON	FAL	VER	11A
PAB 15-65											537						195															89												
PAB 15-67											222						111					2		136						40		1014												
PAB 15-68		41				193		166			938	10												26						5		304											63	
PAB 15-52		24									743						23							62																				
PAB 15-55		345						105			113		43				19		69			74		3						238														
PAB 15-56											161		260				328													45								288						
PAB 15-58		83						7			431		38				161													36		152						196						
PAB 15-80		49			54			68			163		32				255							56					32	30		36												
PAB 15-79								97	50		57		26				107															110											893	
PAB 14-104			977		55																																							
PAB 15-34											2		80																	520														
PAB 15-44											263		71				37													15		285				21		20						
PAB 15-45		101									512						26													66		13						47						
PAB 14-12						2			23				1243	353																			31											
PAB 14-14																																											6	2032
PAB 14-15											1939																																	
PAB 14-18											168					2	525																					690					700	
PAB 15-33											477													195								111												
PAB 14-86													277				504									89	155														96			
PAB 15-70		34						292			223		7		2		457		79					14						14		84												
PAB 15-71		231				110					675						43		79											146		39												
PAB 15-72											737		26									3										233												
PAB 15-73								60			121								671			2								80		18												
PAB 15-77			554					168			73		118	325			15		44													36						60						
PAB 15-78		16								10	30																			26		101												
PAB 15-89		81						71			179						91		126													90												
PAB 15-90											512		86				179													340														

Sample	CAC	NIC	ID	2A	2B	CAP	CAPS	CIT	ELO	FRI	MULT	PAC	ARE	BOO	PAL	PEC	AMN	BIL	CRA	FLU	GON	GRE	INU	KWO	LAC	LIT	MOY	ROS	WAL	THE	CAM	CIN	FRA	NIE	CRY	DRE	ERY	PSE	KEL	8C	CON	FAL	VER	11A
PAB 16-16								5			91			8	7								6		13													61						
PAB 15-105		9			159			68					88				252													28		91												
PAB 15-51		114						2			789	52	74				61															97		188				44						
PAB 15-53		177									98		194				217													69		97												
PAB 14-73						47					1104		26		27									76						87		47						42						
PAB 14-75																											1401																	
PAB 15-35											339		112				127															34												
PAB 15-36		84									42		14				105							115								217												
PAB 15-54											634													137						90		43						21						
PAB 15-57		100									1339		15		4															3		149						5						
PAB 15-37					2						549		41											1226																				
PAB 15-38											2						433							22						182														
PAB 15-20				30	225									510			12															65						77						
PAB 15-21											413	525	20				527							544						311		295												
PAB 15-104					915								161																															525
PAB 15-103		153									22		5				7															5						3					289	
PAB 15-81		55						78			116		19				49													21														
PAB 15-82								127			5						7		7											20		28						12					853	
PAB 15-83		89						127			236	20	45				63													21		19						103						
PAB 15-84		151						175			628		8				161		25					32						243								152						
PAB 15-30											156		12																	46		379												
PAB 15-32		119									195		10											238						134														
PAB 15-66		116									807		4				26							214						117														
PAB 15-47											575		58				84	15														20												
PAB 15-40											203	15	327	120			90							56						41		34												
PAB 15-42													410				9							8								137												
PAB 15-46		13											748	20			33													17		101												

Sample	CAC	NIC	ID	2A	2B	CAP	CAPS	CIT	ELO	FRI	MULT	PAC	ARE	BOO	PAL	PEC	AMN	BIL	CRA	FLU	GON	GRE
PAB 15-39		28									141		325									33
PAB 16-27		6		75							212											
PAB 16-32		4		6							69			13				12				
PAB 15-88		281									270		40		61		98		179			

Sample	INU	KWO	LAC	LIT	MOY	ROS	WAL	THE	CAM	CIN	FRA	NIE	CRY	DRE	ERY	PSE	KEL	8C	CON	FAL	VER	11A
PAB 15-39		31						56		173						124						
PAB 16-27								42			13					110			2			
PAB 16-32	18					4		12		2						36					279	
PAB 15-88					223					62												

Table S3. Co-occurrence species matrix

sp1	sp2	sp1_inc	sp2_inc	obs_cooccur	prob_cooccur	exp_cooccur	p_lt	p_gt	sp1_name	sp2_name
11	13	130	98	84	0.451	75.8	0.99938	0.00215	multivora	arenaria
11	17	130	88	78	0.405	68.1	0.99995	0.00022	multivora	amnicola
11	32	130	81	72	0.373	62.7	0.9999	0.00046	multivora	cinnamomi
11	38	130	77	66	0.355	59.6	0.9953	0.01345	multivora	pseudocryptogea
2	11	70	130	61	0.322	54.2	0.99754	0.00788	nicotianae	multivora
11	30	130	69	61	0.318	53.4	0.99915	0.00315	multivora	thermophila
13	17	98	88	66	0.306	51.3	1	0	arenaria	amnicola
13	32	98	81	58	0.281	47.2	0.99981	0.00062	arenaria	cinnamomi
13	38	98	77	54	0.267	44.9	0.99878	0.00336	arenaria	pseudocryptogea
17	32	88	81	58	0.253	42.4	1	0	amnicola	cinnamomi
2	13	70	98	50	0.243	40.8	0.99903	0.00278	nicotianae	arenaria
13	30	98	69	46	0.24	40.2	0.97706	0.04699	arenaria	thermophila
17	38	88	77	44	0.24	40.3	0.90189	0.16312	amnicola	pseudocryptogea
32	38	81	77	41	0.221	37.1	0.91248	0.14781	cinnamomi	pseudocryptogea
2	17	70	88	50	0.218	36.7	0.99999	2.00E-05	nicotianae	amnicola
17	30	88	69	43	0.215	36.1	0.98977	0.0227	amnicola	thermophila
2	32	70	81	44	0.201	33.8	0.99964	0.00109	nicotianae	cinnamomi
30	32	69	81	43	0.198	33.3	0.99937	0.00182	thermophila	cinnamomi
2	38	70	77	37	0.191	32.1	0.95559	0.08269	nicotianae	pseudocryptogea
30	38	69	77	37	0.188	31.6	0.96782	0.06246	thermophila	pseudocryptogea
2	30	70	69	41	0.171	28.8	0.99998	9.00E-05	nicotianae	thermophila
11	24	130	35	32	0.161	27.1	0.99614	0.01724	multivora	kwongonina
8	11	28	130	25	0.129	21.7	0.97815	0.07469	citrophthora	multivora
13	24	98	35	25	0.122	20.4	0.97655	0.05627	arenaria	kwongonina
6	11	25	130	24	0.115	19.3	0.99909	0.00909	capensis	multivora
17	24	88	35	22	0.109	18.3	0.94423	0.11389	amnicola	kwongonina
11	14	130	23	16	0.106	17.8	0.23759	0.88872	multivora	boodjera
5	11	22	130	11	0.101	17	0.00228	0.99955	AUS2B	multivora
24	32	35	81	24	0.1	16.9	0.99827	0.00566	kwongonina	cinnamomi
8	13	28	98	22	0.097	16.3	0.99629	0.01319	citrophthora	arenaria
24	38	35	77	12	0.095	16	0.08777	0.95937	kwongonina	pseudocryptogea
2	24	70	35	17	0.087	14.6	0.86923	0.22937	nicotianae	kwongonina
6	13	25	98	19	0.087	14.6	0.98677	0.04011	capensis	arenaria
8	17	28	88	24	0.087	14.7	0.99999	7.00E-05	citrophthora	amnicola
24	30	35	69	23	0.086	14.4	0.99978	9.00E-04	kwongonina	thermophila
11	23	130	18	18	0.083	13.9	1	0.00741	multivora	inundata
8	32	28	81	20	0.08	13.5	0.99833	0.00612	citrophthora	cinnamomi
13	14	98	23	13	0.08	13.4	0.51135	0.66423	arenaria	boodjera
6	17	25	88	19	0.078	13.1	0.99781	0.00852	capensis	amnicola
5	13	22	98	14	0.076	12.8	0.77876	0.38227	AUS2B	arenaria
8	38	28	77	15	0.076	12.8	0.86597	0.24394	citrophthora	pseudocryptogea

sp1	sp2	sp1_inc	sp2_inc	obs_cooccur	prob_cooccur	exp_cooccur	p_lt	p_gt	sp1_name	sp2_name
11	15	130	16	16	0.074	12.4	1	0.0132	multivora	palmivora
11	43	130	16	14	0.074	12.4	0.91702	0.24991	multivora	versiformis
6	32	25	81	17	0.072	12.1	0.99138	0.02638	capensis	cinnamomi
14	17	23	88	13	0.072	12	0.74215	0.42067	boodjera	amnicola
2	8	70	28	20	0.069	11.7	0.9999	0.00052	nicotianae	citrophthora
5	17	22	88	13	0.069	11.5	0.81693	0.32864	AUS2B	amnicola
11	12	130	15	13	0.069	11.6	0.89616	0.29485	multivora	pachypleura
11	19	130	15	12	0.069	11.6	0.70515	0.54736	multivora	crassamura
11	27	130	15	12	0.069	11.6	0.70515	0.54736	multivora	moyootj
6	38	25	77	18	0.068	11.5	0.99901	0.00412	capensis	pseudocryptogea
8	30	28	69	17	0.068	11.5	0.99394	0.01828	citrophthora	thermophila
14	32	23	81	14	0.066	11.1	0.93758	0.13944	boodjera	cinnamomi
11	18	130	14	13	0.064	10.8	0.97669	0.12931	multivora	bilborang
11	22	130	14	14	0.064	10.8	1	0.02331	multivora	gregata
5	32	22	81	13	0.063	10.6	0.90744	0.19322	AUS2B	cinnamomi
14	38	23	77	15	0.063	10.5	0.98744	0.03726	boodjera	pseudocryptogea
2	6	70	25	14	0.062	10.4	0.96297	0.08828	nicotianae	capensis
13	23	98	18	12	0.062	10.5	0.84421	0.31002	arenaria	inundata
6	30	25	69	12	0.061	10.3	0.83741	0.29165	capensis	thermophila
5	38	22	77	10	0.06	10.1	0.57742	0.60353	AUS2B	pseudocryptogea
11	28	130	13	10	0.06	10.1	0.59863	0.66669	multivora	rosacearum
2	14	70	23	11	0.057	9.6	0.80901	0.33577	nicotianae	boodjera
13	15	98	16	10	0.056	9.3	0.72981	0.46964	arenaria	palmivora
13	43	98	16	9	0.056	9.3	0.53036	0.67454	arenaria	versiformis
14	30	23	69	15	0.056	9.4	0.99699	0.01101	boodjera	thermophila
17	23	88	18	10	0.056	9.4	0.70236	0.4871	amnicola	inundata
2	5	70	22	10	0.055	9.2	0.73347	0.43501	nicotianae	AUS2B
4	11	12	130	10	0.055	9.3	0.80272	0.4626	AUS2A	multivora
5	30	22	69	11	0.054	9	0.87359	0.24661	AUS2B	thermophila
12	13	15	98	11	0.052	8.8	0.93777	0.16893	pachypleura	arenaria
13	19	98	15	7	0.052	8.8	0.24478	0.89087	arenaria	crassamura
13	27	98	15	13	0.052	8.8	0.99744	0.01604	arenaria	moyootj
23	32	18	81	10	0.052	8.7	0.81828	0.34066	inundata	cinnamomi
11	41	130	11	9	0.051	8.5	0.7585	0.52838	multivora	constricta
15	17	16	88	9	0.05	8.4	0.72062	0.47652	palmivora	amnicola
17	43	88	16	11	0.05	8.4	0.95151	0.13206	amnicola	versiformis
13	18	98	14	9	0.049	8.2	0.77239	0.4307	arenaria	bilborang
13	22	98	14	9	0.049	8.2	0.77239	0.4307	arenaria	gregata
23	38	18	77	17	0.049	8.2	1	1.00E-05	inundata	pseudocryptogea
12	17	15	88	11	0.047	7.9	0.97797	0.07461	pachypleura	amnicola
17	19	88	15	10	0.047	7.9	0.92539	0.18731	amnicola	crassamura
17	27	88	15	13	0.047	7.9	0.99943	0.00451	amnicola	moyootj

sp1	sp2	sp1_inc	sp2_inc	obs_cooccur	prob_cooccur	exp_cooccur	p_lt	p_gt	sp1_name	sp2_name
11	26	130	10	9	0.046	7.7	0.92908	0.29366	multivora	litoralis
15	32	16	81	10	0.046	7.7	0.9291	0.17393	palmivora	cinnamomi
32	43	81	16	12	0.046	7.7	0.99486	0.02224	cinnamomi	versiformis
2	23	70	18	14	0.045	7.5	0.99982	0.00119	nicotianae	inundata
13	28	98	13	9	0.045	7.6	0.8702	0.30015	arenaria	rosacearum
15	38	16	77	13	0.044	7.3	0.99959	0.00283	palmivora	pseudocryptogea
17	18	88	14	5	0.044	7.3	0.15285	0.94403	amnicola	bilborang
17	22	88	14	9	0.044	7.3	0.88783	0.25864	amnicola	gregata
23	30	18	69	13	0.044	7.4	0.99901	0.00499	inundata	thermophila
38	43	77	16	12	0.044	7.3	0.99717	0.0135	pseudocryptogea	versiformis
12	32	15	81	10	0.043	7.2	0.96255	0.10953	pachypleura	cinnamomi
19	32	15	81	9	0.043	7.2	0.89047	0.24637	crassamura	cinnamomi
27	32	15	81	8	0.043	7.2	0.75363	0.44157	moyootj	cinnamomi
4	13	12	98	6	0.042	7	0.37617	0.81936	AUS2A	arenaria
3	11	9	130	6	0.041	7	0.33118	0.88177	AUS1D	multivora
12	38	15	77	9	0.041	6.9	0.92297	0.18872	pachypleura	pseudocryptogea
17	28	88	13	8	0.041	6.8	0.83552	0.34691	amnicola	rosacearum
19	38	15	77	6	0.041	6.9	0.42212	0.77089	crassamura	pseudocryptogea
27	38	15	77	11	0.041	6.9	0.9945	0.02405	moyootj	pseudocryptogea
2	15	70	16	10	0.04	6.7	0.97897	0.06638	nicotianae	palmivora
2	43	70	16	10	0.04	6.7	0.97897	0.06638	nicotianae	versiformis
18	32	14	81	7	0.04	6.7	0.66254	0.55412	bilborang	cinnamomi
22	32	14	81	9	0.04	6.7	0.93852	0.16427	gregata	cinnamomi
15	30	16	69	8	0.039	6.6	0.84846	0.30702	palmivora	thermophila
30	43	69	16	8	0.039	6.6	0.84846	0.30702	thermophila	versiformis
13	41	98	11	10	0.038	6.4	0.99792	0.02025	arenaria	constricta
18	38	14	77	11	0.038	6.4	0.99821	0.01045	bilborang	pseudocryptogea
22	38	14	77	9	0.038	6.4	0.95829	0.12171	gregata	pseudocryptogea
2	12	70	15	6	0.037	6.2	0.55929	0.65502	nicotianae	pachypleura
2	19	70	15	9	0.037	6.2	0.96197	0.10913	nicotianae	crassamura
2	27	70	15	8	0.037	6.2	0.89087	0.24478	nicotianae	moyootj
4	17	12	88	5	0.037	6.3	0.31844	0.85787	AUS2A	amnicola
11	33	130	8	7	0.037	6.2	0.87777	0.42431	multivora	fragariae
12	30	15	69	7	0.037	6.2	0.77072	0.42112	pachypleura	thermophila
19	30	15	69	7	0.037	6.2	0.77072	0.42112	crassamura	thermophila
27	30	15	69	7	0.037	6.2	0.77072	0.42112	moyootj	thermophila
28	32	13	81	8	0.037	6.3	0.90181	0.23849	rosacearum	cinnamomi
2	18	70	14	9	0.035	5.8	0.98062	0.06647	nicotianae	bilborang
2	22	70	14	9	0.035	5.8	0.98062	0.06647	nicotianae	gregata
8	24	28	35	8	0.035	5.8	0.90966	0.19511	citrophthora	kwongonina
13	26	98	10	10	0.035	5.8	1	0.00373	arenaria	litoralis
28	38	13	77	10	0.035	6	0.99635	0.01938	rosacearum	pseudocryptogea

sp1	sp2	sp1_inc	sp2_inc	obs_cooccur	prob_cooccur	exp_cooccur	p_lt	p_gt	sp1_name	sp2_name
4	32	12	81	7	0.034	5.8	0.84785	0.33417	AUS2A	cinnamomi
17	41	88	11	9	0.034	5.8	0.99271	0.04075	amnicola	constricta
18	30	14	69	11	0.034	5.7	0.9995	0.00355	bilborang	thermophila
22	30	14	69	11	0.034	5.7	0.9995	0.00355	gregata	thermophila
4	38	12	77	11	0.033	5.5	0.99995	0.00091	AUS2A	pseudocryptogea
2	28	70	13	7	0.032	5.4	0.88811	0.26086	nicotianae	rosacearum
11	39	130	7	5	0.032	5.4	0.49682	0.80794	multivora	sp. kelmania
28	30	13	69	8	0.032	5.3	0.96737	0.10324	rosacearum	thermophila
32	41	81	11	6	0.032	5.3	0.77201	0.45026	cinnamomi	constricta
3	13	9	98	5	0.031	5.2	0.56137	0.70221	AUS1D	arenaria
6	24	25	35	5	0.031	5.2	0.57595	0.63456	capensis	kwongonina
17	26	88	10	9	0.031	5.2	0.9988	0.01337	amnicola	litoralis
2	4	70	12	8	0.03	5	0.983	0.06531	nicotianae	AUS2A
38	41	77	11	10	0.03	5	0.99988	0.00197	pseudocryptogea	constricta
4	30	12	69	10	0.029	4.9	0.99973	0.00261	AUS2A	thermophila
14	24	23	35	3	0.029	4.8	0.24457	0.90313	boodjera	kwongonina
26	32	10	81	5	0.029	4.8	0.67098	0.58107	litoralis	cinnamomi
3	17	9	88	4	0.028	4.7	0.44014	0.79716	AUS1D	amnicola
9	11	6	130	4	0.028	4.6	0.40974	0.87014	elongata	multivora
13	33	98	8	7	0.028	4.7	0.98816	0.08472	arenaria	fragariae
2	41	70	11	6	0.027	4.6	0.88663	0.2784	nicotianae	constricta
5	24	22	35	8	0.027	4.6	0.98214	0.05551	AUS2B	kwongonina
26	38	10	77	8	0.027	4.6	0.9958	0.02714	litoralis	pseudocryptogea
30	41	69	11	6	0.027	4.5	0.89466	0.26426	thermophila	constricta
3	32	9	81	3	0.026	4.3	0.28473	0.89765	AUS1D	cinnamomi
2	26	70	10	5	0.025	4.2	0.81162	0.4072	nicotianae	litoralis
3	38	9	77	5	0.025	4.1	0.8276	0.39593	AUS1D	pseudocryptogea
6	8	25	28	6	0.025	4.2	0.90834	0.21357	capensis	citrophthora
17	33	88	8	5	0.025	4.2	0.82807	0.41415	amnicola	fragariae
13	39	98	7	3	0.024	4.1	0.3196	0.8916	arenaria	sp. kelmania
26	30	10	69	4	0.024	4.1	0.60843	0.64947	litoralis	thermophila
8	14	28	23	4	0.023	3.8	0.67067	0.55989	citrophthora	boodjera
11	31	130	5	4	0.023	3.9	0.72744	0.68355	multivora	cambivora
32	33	81	8	5	0.023	3.9	0.88345	0.32092	cinnamomi	fragariae
2	3	70	9	2	0.022	3.8	0.19472	0.94671	nicotianae	AUS1D
3	30	9	69	4	0.022	3.7	0.71543	0.54631	AUS1D	thermophila
5	8	22	28	6	0.022	3.7	0.95239	0.13175	AUS2B	citrophthora
17	39	88	7	1	0.022	3.7	0.04486	0.99519	amnicola	sp. kelmania
23	24	18	35	3	0.022	3.8	0.45833	0.77177	inundata	kwongonina
33	38	8	77	7	0.022	3.7	0.99842	0.01805	fragariae	pseudocryptogea
9	13	6	98	5	0.021	3.5	0.96311	0.20351	elongata	arenaria
2	33	70	8	6	0.02	3.3	0.99045	0.05645	nicotianae	fragariae

sp1	sp2	sp1_inc	sp2_inc	obs_cooccur	prob_cooccur	exp_cooccur	p_lt	p_gt	sp1_name	sp2_name
6	14	25	23	5	0.02	3.4	0.90056	0.23937	capensis	boodjera
15	24	16	35	3	0.02	3.3	0.56333	0.69048	palmivora	kwongonina
24	43	35	16	5	0.02	3.3	0.91452	0.21852	kwongonina	versiformis
30	33	69	8	6	0.02	3.3	0.99133	0.05243	thermophila	fragariae
32	39	81	7	0	0.02	3.4	0.00885	1	cinnamomi	sp. kelmania
5	6	22	25	4	0.019	3.3	0.79105	0.42044	AUS2B	capensis
9	17	6	88	5	0.019	3.1	0.98101	0.12884	elongata	amnicola
12	24	15	35	7	0.019	3.1	0.99639	0.01729	pachypleura	kwongonina
19	24	15	35	3	0.019	3.1	0.61787	0.64289	crassamura	kwongonina
24	27	35	15	3	0.019	3.1	0.61787	0.64289	kwongonina	moyootj
38	39	77	7	3	0.019	3.2	0.59216	0.70393	pseudocryptogea	sp. kelmania
5	14	22	23	5	0.018	3	0.94363	0.1596	AUS2B	boodjera
8	23	28	18	3	0.018	3	0.6504	0.60899	citrophthora	inundata
10	11	4	130	4	0.018	3.1	1	0.35476	frigida	multivora
2	39	70	7	3	0.017	2.9	0.6804	0.61907	nicotianae	sp. kelmania
9	32	6	81	4	0.017	2.9	0.9105	0.30765	elongata	cinnamomi
13	31	98	5	4	0.017	2.9	0.93534	0.30543	arenaria	cambivora
18	24	14	35	1	0.017	2.9	0.16588	0.96737	bilborang	kwongonina
22	24	14	35	4	0.017	2.9	0.86098	0.32741	gregata	kwongonina
30	39	69	7	3	0.017	2.9	0.69247	0.60624	thermophila	sp. kelmania
6	23	25	18	7	0.016	2.7	0.99871	0.00731	capensis	inundata
8	15	28	16	3	0.016	2.7	0.73529	0.52005	citrophthora	palmivora
8	43	28	16	5	0.016	2.7	0.96974	0.10263	citrophthora	versiformis
9	38	6	77	3	0.016	2.7	0.73444	0.57703	elongata	pseudocryptogea
17	31	88	5	4	0.016	2.6	0.9627	0.21493	amnicola	cambivora
24	28	35	13	3	0.016	2.7	0.72641	0.53431	kwongonina	rosacearum
2	9	70	6	3	0.015	2.5	0.80109	0.49073	nicotianae	elongata
4	24	12	35	0	0.015	2.5	0.05434	1	AUS2A	kwongonina
8	12	28	15	3	0.015	2.5	0.77581	0.47217	citrophthora	pachypleura
8	19	28	15	8	0.015	2.5	0.99993	0.00065	citrophthora	crassamura
8	27	28	15	7	0.015	2.5	0.99935	0.00432	citrophthora	moyootj
9	30	6	69	3	0.015	2.5	0.80981	0.47822	elongata	thermophila
14	23	23	18	8	0.015	2.5	0.99992	0.00066	boodjera	inundata
5	23	22	18	2	0.014	2.4	0.57096	0.71953	AUS2B	inundata
6	15	25	16	4	0.014	2.4	0.93318	0.19732	capensis	palmivora
6	43	25	16	6	0.014	2.4	0.99675	0.01699	capensis	versiformis
8	18	28	14	0	0.014	2.3	0.06941	1	citrophthora	bilborang
8	22	28	14	2	0.014	2.3	0.57743	0.71633	citrophthora	gregata
10	13	4	98	2	0.014	2.3	0.55467	0.80382	frigida	arenaria
11	35	130	3	3	0.014	2.3	1	0.4609	multivora	cryptogea
11	44	130	3	1	0.014	2.3	0.12861	0.98913	multivora	AUS11A
24	41	35	11	3	0.014	2.3	0.82662	0.41107	kwongonina	constricta

sp1	sp2	sp1_inc	sp2_inc	obs_cooccur	prob_cooccur	exp_cooccur	p_lt	p_gt	sp1_name	sp2_name
31	32	5	81	3	0.014	2.4	0.83778	0.46614	cambivora	cinnamomi
31	38	5	77	4	0.014	2.3	0.98119	0.13607	cambivora	pseudocryptogea
6	12	25	15	5	0.013	2.2	0.98816	0.05125	capensis	pachypleura
6	19	25	15	2	0.013	2.2	0.6084	0.68983	capensis	crassamura
6	27	25	15	8	0.013	2.2	0.99998	0.00026	capensis	moyootj
8	28	28	13	4	0.013	2.2	0.95589	0.14996	citrophthora	rosacearum
14	15	23	16	5	0.013	2.2	0.98911	0.04786	boodjera	palmivora
14	43	23	16	3	0.013	2.2	0.84349	0.37883	boodjera	versiformis
2	31	70	5	5	0.012	2.1	1	0.01152	nicotianae	cambivora
4	8	12	28	0	0.012	2	0.10325	1	AUS2A	citrophthora
5	12	22	15	4	0.012	2	0.96996	0.11317	AUS2B	pachypleura
5	15	22	16	0	0.012	2.1	0.09432	1	AUS2B	palmivora
5	19	22	15	0	0.012	2	0.11016	1	AUS2B	crassamura
5	27	22	15	4	0.012	2	0.96996	0.11317	AUS2B	moyootj
5	43	22	16	3	0.012	2.1	0.86239	0.34983	AUS2B	versiformis
6	18	25	14	2	0.012	2.1	0.65378	0.65007	capensis	bilborang
6	22	25	14	3	0.012	2.1	0.86556	0.34622	capensis	gregata
6	28	25	13	6	0.012	1.9	0.99933	0.00504	capensis	rosacearum
10	17	4	88	3	0.012	2.1	0.92717	0.34701	frigida	amnicola
12	14	15	23	4	0.012	2.1	0.96369	0.12935	pachypleura	boodjera
14	19	23	15	3	0.012	2.1	0.87065	0.33714	boodjera	crassamura
14	27	23	15	2	0.012	2.1	0.66286	0.64084	boodjera	moyootj
24	26	35	10	1	0.012	2.1	0.3434	0.91017	kwongonina	litoralis
30	31	69	5	3	0.012	2.1	0.90781	0.3342	thermophila	cambivora
3	24	9	35	0	0.011	1.9	0.11519	1	AUS1D	kwongonina
4	6	12	25	2	0.011	1.8	0.74391	0.55961	AUS2A	capensis
5	18	22	14	0	0.011	1.8	0.12852	1	AUS2B	bilborang
5	22	22	14	1	0.011	1.8	0.42615	0.87148	AUS2B	gregata
8	41	28	11	4	0.011	1.8	0.97964	0.08852	citrophthora	constricta
10	32	4	81	4	0.011	1.9	1	0.05196	frigida	cinnamomi
10	38	4	77	3	0.011	1.8	0.95774	0.25016	frigida	pseudocryptogea
14	18	23	14	7	0.011	1.9	0.99993	0.00067	boodjera	bilborang
14	22	23	14	3	0.011	1.9	0.89546	0.29558	boodjera	gregata
14	28	23	13	2	0.011	1.8	0.74536	0.55681	boodjera	rosacearum
2	10	70	4	4	0.01	1.7	1	0.02864	nicotianae	frigida
4	14	12	23	8	0.01	1.6	1	1.00E-05	AUS2A	boodjera
5	28	22	13	4	0.01	1.7	0.9845	0.07128	AUS2B	rosacearum
6	41	25	11	7	0.01	1.6	0.99999	0.00016	capensis	constricta
8	26	28	10	4	0.01	1.7	0.9874	0.06374	citrophthora	litoralis
10	30	4	69	4	0.01	1.6	1	0.027	frigida	thermophila
12	23	15	18	1	0.01	1.6	0.50351	0.83134	pachypleura	inundata
13	35	98	3	3	0.01	1.8	1	0.19595	arenaria	cryptogea

sp1	sp2	sp1_inc	sp2_inc	obs_cooccur	prob_cooccur	exp_cooccur	p_lt	p_gt	sp1_name	sp2_name
13	44	98	3	1	0.01	1.8	0.37542	0.92948	arenaria	AUS11A
15	23	16	18	5	0.01	1.7	0.99733	0.01652	palmivora	inundata
19	23	15	18	2	0.01	1.6	0.79437	0.49649	crassamura	inundata
23	27	18	15	2	0.01	1.6	0.79437	0.49649	inundata	moyootj
23	43	18	16	4	0.01	1.7	0.98348	0.07383	inundata	versiformis
24	33	35	8	2	0.01	1.7	0.78211	0.52491	kwongonina	fragariae
1	11	2	130	1	0.009	1.5	0.40227	0.94989	cactorum	multivora
3	8	9	28	2	0.009	1.5	0.82662	0.46075	AUS1D	citrophthora
4	5	12	22	1	0.009	1.6	0.51537	0.82563	AUS2A	AUS2B
5	41	22	11	4	0.009	1.4	0.99331	0.03956	AUS2B	constricta
6	26	25	10	7	0.009	1.5	1	6.00E-05	capensis	litoralis
11	25	130	2	2	0.009	1.5	1	0.59773	multivora	lacustris
11	34	130	2	2	0.009	1.5	1	0.59773	multivora	niederhauserii
11	36	130	2	2	0.009	1.5	1	0.59773	multivora	drechsleri
11	37	130	2	2	0.009	1.5	1	0.59773	multivora	erythroseptica
11	40	130	2	2	0.009	1.5	1	0.59773	multivora	AUS8C
12	15	15	16	1	0.009	1.4	0.56911	0.79223	pachypleura	palmivora
12	43	15	16	3	0.009	1.4	0.96035	0.15793	pachypleura	versiformis
14	41	23	11	2	0.009	1.5	0.82315	0.46108	boodjera	constricta
15	19	16	15	2	0.009	1.4	0.84207	0.43089	palmivora	crassamura
15	27	16	15	2	0.009	1.4	0.84207	0.43089	palmivora	moyootj
15	43	16	16	1	0.009	1.5	0.53369	0.81396	palmivora	versiformis
17	35	88	3	2	0.009	1.6	0.85863	0.5359	amnicola	cryptogea
17	44	88	3	0	0.009	1.6	0.10585	1	amnicola	AUS11A
18	23	14	18	9	0.009	1.5	1	0	bilborang	inundata
19	43	15	16	2	0.009	1.4	0.84207	0.43089	crassamura	versiformis
22	23	14	18	5	0.009	1.5	0.99885	0.00873	gregata	inundata
24	39	35	7	0	0.009	1.5	0.1884	1	kwongonina	sp. kelmania
27	43	15	16	4	0.009	1.4	0.99306	0.03965	moyootj	versiformis
32	35	81	3	3	0.009	1.4	1	0.10992	cinnamomi	cryptogea
32	44	81	3	0	0.009	1.4	0.13655	1	cinnamomi	AUS11A
3	6	9	25	2	0.008	1.3	0.86703	0.39839	AUS1D	capensis
4	23	12	18	7	0.008	1.3	1	3.00E-05	AUS2A	inundata
5	26	22	10	1	0.008	1.3	0.61392	0.76441	AUS2B	litoralis
8	33	28	8	3	0.008	1.3	0.97278	0.13001	citrophthora	fragariae
12	19	15	15	0	0.008	1.3	0.23035	1	pachypleura	crassamura
12	27	15	15	0	0.008	1.3	0.23035	1	pachypleura	moyootj
14	26	23	10	2	0.008	1.4	0.85879	0.4094	boodjera	litoralis
15	18	16	14	4	0.008	1.3	0.99509	0.03093	palmivora	bilborang
15	22	16	14	2	0.008	1.3	0.86572	0.39449	palmivora	gregata
18	43	14	16	1	0.008	1.3	0.60551	0.76814	bilborang	versiformis
19	27	15	15	2	0.008	1.3	0.86424	0.39677	crassamura	moyootj

sp1	sp2	sp1_inc	sp2_inc	obs_cooccur	prob_cooccur	exp_cooccur	p_lt	p_gt	sp1_name	sp2_name
22	43	14	16	0	0.008	1.3	0.23186	1	gregata	versiformis
23	28	18	13	4	0.008	1.4	0.99398	0.03598	inundata	rosacearum
35	38	3	77	2	0.008	1.4	0.90576	0.43727	cryptogea	pseudocryptogea
38	44	77	3	1	0.008	1.4	0.56273	0.84349	pseudocryptogea	AUS11A
1	13	2	98	1	0.007	1.2	0.66118	0.82784	cactorum	arenaria
2	35	70	3	3	0.007	1.2	1	0.07052	nicotianae	cryptogea
2	44	70	3	1	0.007	1.2	0.62458	0.80405	nicotianae	AUS11A
3	5	9	22	2	0.007	1.2	0.90281	0.33427	AUS1D	AUS2B
3	14	9	23	2	0.007	1.2	0.89144	0.35577	AUS1D	boodjera
4	15	12	16	4	0.007	1.1	0.99781	0.0173	AUS2A	palmivora
4	43	12	16	1	0.007	1.1	0.6804	0.7119	AUS2A	versiformis
6	33	25	8	7	0.007	1.2	1	1.00E-05	capensis	fragariae
8	39	28	7	0	0.007	1.2	0.27199	1	citrophthora	sp. kelmania
9	24	6	35	1	0.007	1.2	0.6346	0.75968	elongata	kwongonina
12	18	15	14	0	0.007	1.2	0.25521	1	pachypleura	bilborang
12	22	15	14	0	0.007	1.2	0.25521	1	pachypleura	gregata
12	28	15	13	0	0.007	1.2	0.28256	1	pachypleura	rosacearum
13	25	98	2	1	0.007	1.2	0.66118	0.82784	arenaria	lacustris
13	34	98	2	2	0.007	1.2	1	0.33882	arenaria	niederhauserii
13	36	98	2	2	0.007	1.2	1	0.33882	arenaria	drechsleri
13	37	98	2	1	0.007	1.2	0.66118	0.82784	arenaria	erythroseptica
13	40	98	2	1	0.007	1.2	0.66118	0.82784	arenaria	AUS8C
14	33	23	8	3	0.007	1.1	0.98689	0.07951	boodjera	fragariae
15	28	16	13	0	0.007	1.2	0.25855	1	palmivora	rosacearum
18	19	14	15	1	0.007	1.2	0.63803	0.74479	bilborang	crassamura
18	22	14	14	5	0.007	1.2	0.99978	0.00248	bilborang	gregata
18	27	14	15	0	0.007	1.2	0.25521	1	bilborang	moyootj
19	22	15	14	2	0.007	1.2	0.8851	0.36197	crassamura	gregata
19	28	15	13	1	0.007	1.2	0.67333	0.71744	crassamura	rosacearum
22	27	14	15	1	0.007	1.2	0.63803	0.74479	gregata	moyootj
23	41	18	11	3	0.007	1.2	0.98083	0.09868	inundata	constricta
27	28	15	13	6	0.007	1.2	0.99999	0.00021	moyootj	rosacearum
28	43	13	16	4	0.007	1.2	0.99665	0.02349	rosacearum	versiformis
30	35	69	3	1	0.007	1.2	0.63329	0.79793	thermophila	cryptogea
30	44	69	3	0	0.007	1.2	0.20207	1	thermophila	AUS11A
1	17	2	88	0	0.006	1	0.22526	1	cactorum	amnicola
4	12	12	15	0	0.006	1.1	0.31262	1	AUS2A	pachypleura
4	18	12	14	10	0.006	1	1	0	AUS2A	bilborang
4	19	12	15	1	0.006	1.1	0.70889	0.68738	AUS2A	crassamura
4	22	12	14	4	0.006	1	0.99892	0.01033	AUS2A	gregata
4	27	12	15	0	0.006	1.1	0.31262	1	AUS2A	moyootj
5	33	22	8	2	0.006	1	0.92915	0.28159	AUS2B	fragariae

sp1	sp2	sp1_inc	sp2_inc	obs_cooccur	prob_cooccur	exp_cooccur	p_lt	p_gt	sp1_name	sp2_name
6	39	25	7	0	0.006	1	0.31653	1	capensis	sp. kelmania
8	9	28	6	2	0.006	1	0.94113	0.26197	citrophthora	elongata
15	41	16	11	0	0.006	1	0.32079	1	palmivora	constricta
17	25	88	2	1	0.006	1	0.72712	0.77474	amnicola	lacustris
17	34	88	2	1	0.006	1	0.72712	0.77474	amnicola	niederhauserii
17	36	88	2	2	0.006	1	1	0.27288	amnicola	drechsleri
17	37	88	2	1	0.006	1	0.72712	0.77474	amnicola	erythroseptica
17	40	88	2	1	0.006	1	0.72712	0.77474	amnicola	AUS8C
18	28	14	13	2	0.006	1.1	0.9199	0.29585	bilborang	rosacearum
22	28	14	13	2	0.006	1.1	0.9199	0.29585	gregata	rosacearum
23	26	18	10	2	0.006	1.1	0.92301	0.29114	inundata	litoralis
24	31	35	5	1	0.006	1	0.72119	0.69397	kwongonina	cambivora
41	43	11	16	5	0.006	1	0.9999	0.00135	constricta	versiformis
